# Poly(Vinyl Alcohol) Cryogel Membranes Loaded with Resveratrol as Potential Active Wound Dressings

**DOI:** 10.1208/s12249-021-01976-1

**Published:** 2021-03-14

**Authors:** Anna Górska, Anna Krupa, Dorota Majda, Piotr Kulinowski, Mateusz Kurek, Władysław P. Węglarz, Renata Jachowicz

**Affiliations:** 1 Department of Pharmaceutical Technology and Biopharmaceutics, Faculty of Pharmacy, Jagiellonian University Collegium Medicum, 9 Medyczna Street, 30-688 Cracow, Poland; 2 Faculty of Chemistry, Jagiellonian University, Cracow, Poland; 3 Institute of Technology, Pedagogical University of Krakow, Cracow, Poland; 4 Department of Magnetic Resonance Imaging, Institute of Nuclear Physics Polish Academy of Sciences, Cracow, Poland

**Keywords:** wound dressings, wound healing, cryogels, resveratrol, polyvinyl alcohol

## Abstract

Hydrogel wound dressings are highly effective in the therapy of wounds. Yet, most of them do not contain any active ingredient that could accelerate healing. The aim of this study was to prepare hydrophilic active dressings loaded with an anti-inflammatory compound - trans-resveratrol (RSV) of hydrophobic properties. A special attention was paid to select such a technological strategy that could both reduce the risk of irritation at the application site and ensure the homogeneity of the final hydrogel. RSV dissolved in Labrasol was combined with an aqueous sol of poly(vinyl) alcohol (PVA), containing propylene glycol (PG) as a plasticizer. This sol was transformed into a gel under six consecutive cycles of freezing (-80 °C) and thawing (RT). White, uniform and elastic membranes were successfully produced. Their critical features, namely microstructure, mechanical properties, water uptake and RSV release were studied using SEM, DSC, MRI, texture analyser and Franz-diffusion cells. The cryogels made of 8 % of PVA showed optimal tensile strength (0.22 MPa) and elasticity (0.082 MPa). The application of MRI enabled to elucidate mass transport related phenomena in this complex system at the molecular (detection of PG, confinement effects related to pore size) as well as at the macro level (swelling). The controlled release of RSV from membranes was observed for 48 h with mean dissolution time of 18 h and dissolution efficiency of 35 %. All in all, these cryogels could be considered as a promising new active wound dressings.

**Supplementary Information:**

The online version contains supplementary material available at 10.1208/s12249-021-01976-1.

## INTRODUCTION

Under physiological conditions, the wound healing process is due to four overlapping phases, namely, hemostasis, inflammation, proliferation, and remodeling (1). If any of these events is perturbed, the wound fails to heal within a standard three months therapy, often leaving serious scars (2, 3). The number of patients suffering from chronic wounds, and the morbidity associated with them have been rising constantly throughout the recent years (4, 5). A troublesome wound healing can be related to elevated level and prolonged expression of proinflammatory cytokines, high oxidative stress, low oxygen levels, modified expression of proteases and formation of biofilms (6).

The chronic wounds can be treated using hydrogel dressings. The most important advantages of these dressings are nonadherence, tissue-like water content, soft, rubbery consistency, and flexibility that ensure their high tolerance in the wound area (3, 5). After application, they create a beneficial skin microclimate by maintaining a proper moisture balance in the wound bed, which promotes autolytic debridement and gives both pleasant cooling and analgesic effect (3, 5).

The hydrogel membranes have a characteristic three-dimensional (3D) network structure in which pores are created between chains of hydrophilic polymers, e.g., polyvinyl alcohol, alginates, carboxymethylcellulose, and chitosan (7, 8). These pores are filled with water and the diffusion of solutes imbedded in the polymer networks out of the system, toward the wound bed is possible (9, 10). A detailed description of physicochemical properties of PVA hydrogels for both pharmaceutical and medical applications can be found in many excellent original papers and reviews (8, 11–13). In brief, PVA hydrogels can be prepared by chemical or physical cross-linking. The advantage of the latter approach is the lack of chemical intermediates, which can be responsible for serious toxicity and side effects. The physical sol–gel phase transition in PVA systems can be induced by repeated cycles of freezing and thawing (FT). The cryogels formed under such a processing might be used as scaffolds for the repair and the regeneration of various tissues (14). Their high stability, non-adhering properties, elasticity and excellent shape memory make them an interesting option for the modern therapy of wounds located in these parts of the body which are particularly exposed to the mechanical stress (15–17). Moreover, self-healing properties of PVA cryogels were also reported (18).

Since the majority of hydrogel dressings do not contain any active ingredient, this study was focused on the design of physically cross-linked PVA hydrogel membranes that could be a platform for the local delivery of *trans*-resveratrol (RSV, 3,4′,5-trihydroxy-*trans*-stilbene). As far as we know, the solid elastic PVA cryogels have not been used as matrices for the topical administration of RSV with the aim to accelerate the wound healing process. According to many recent studies, this active ingredient showed both an antioxidant and an antiinflammatory effect (19, 20). Moreover, after the application of scaffolds formed using poly (ε-caprolactone) loaded with 0.25–1% of RSV, the expression of the vascular endothelial growth factor was modulated and the matrix metalloproteinases were inhibited (21). Alasmari *et al*. (22) demonstrated that topical application of an ointment, containing 0.5% of RSV stimulated the reepithelialization process and the synthesis of collagen. In turn, Gokce *et al*. (23) developed collagen–laminin dermal matrices impregnated with 0.5–1% RSV loaded in microparticles, which also showed a significant antioxidant effect.

From the physicochemical point of view, RSV is a natural polyphenol of a stiff stilbene structure. There are two structural isomers of RSV: *cis* and *trans* of which *trans*-RSV is both more stable and more biologically active than *cis*-RSV. This compound is sensitive to light and has limited solubility in water [< 1 mg/mL; pKa_1_ = 8.8; pKa_2_ = 9.8; pKa_3_ = 11.4] (24). In consequence, its combination with an aqueous solution of PVA could be challenging, especially if a uniform distribution of RSV within the hydrophilic polymer matrix is required.

Thus, the aim of this research was (1) to design PVA cryogel membranes of controlled release of RSV that could accelerate the healing of chronic wounds and (2) to assess the impact that RSV may have on the properties of such cryogels.

This study was carried out in three main stages. In the first stage, the efforts were made to find a nonirritant and a nontoxic solvent for RSV that could be combined with an aqueous sol of PVA to form a homogeneous system. The second stage relied on preparing PVA cryogels loaded with the solution of RSV in a freezing and thawing technique. In the third stage, the cryogel membranes loaded with RSV were thoroughly characterized. Briefly, the impact of RSV on morphology, microstructure, water uptake, mechanical properties, and release rate was examined in details. Modern analytical tools, i.e., scanning electron microscopy (SEM), texture analysis, differential scanning calorimetry (DSC) or magnetic resonance imaging (MRI) were used to assess the most important features of cryogels loaded with RSV with the aim to assess their functionality as novel wound dressings.

## MATERIALS AND METHODS

*Trans*-resveratrol (RSV, purity >99%) was purchased from Mega Resveratrol (Danbury, CT, USA). Polyvinyl alcohol (PVA, Mowiol® 56–98, Mw ~195.000 Da; degree of hydrolysis ~98.4 ± 0.4mol%) was obtained from Merck (Warsaw, Poland). Propylene glycol (PG) was acquired from Chempur (Piekary Śląskie, Poland). Labrasol (L) was kindly donated by Gattefossé (Saint-Priest, France). Tween 80 was purchased from Croda (Cracow, Poland). Cremophor RH40 was obtained from BASF (Warsaw, Poland). Potassium dihydrogen phosphate, disodium hydrogen phosphate, and sodium chloride were purchased from Avantor Performance Materials (Gliwice, Poland). All these reagents were of the analytical grade.

### Solubility Studies of RSV

The solubility of RSV was determined in (1) pure solvents, namely, distilled water (W), phosphate buffer of pH = 6.8 (PBS, Ph. Eur. 4th ed.), propylene glycol (PG), and Labrasol (L) and (2) a mixture of solvents, i.e., PG and water (1:1). These solvents were chosen taking into account their suitability to prepare wound dressings, i.e., low toxicity and low risk of irritation. An excess amount of RSV was added to a conical flask containing 20 mL of the solvent. The samples, protected from light, were shaken at 37°C for 24 h using a laboratory shaker (Memmert WNB 22, Schwabach, Germany) to reach the saturation. After 24 h, the samples were withdrawn and they were centrifuged (MPW 221, Poland) at 3600 rpm for 30 min. Then, they were filtered through syringe membrane filters of 0.45 μm (Chromafil® Xtra CA-45/25, Macherey-Nagel, Düren, Germany). After dilution, the concentration of RSV was measured spectrophotometrically (Shimadzu UV-1800, Shimadzu USA Manufacturing Inc., Canby, OR, USA) at 306 nm. Mean values (*n*=3) ± standard deviations (SD) were calculated.

### Preparation of Cryogel Membranes

A detailed composition of cryogel membranes was given in Table I. In brief, three kinds of hydrogel membranes, containing 0.4% (*w/w*) of RSV, were prepared using PVA as a matrix-forming agent. The concentration of PVA in the cryogels was equal to 5, 8, or 10% (*w/w*).

**Table I:** Composition of hydrogel membranes (% *w/w*)

Formulation name	% of PVA in matrix	RSV [%]	L [%]	PVA [%]	PG [%]	W [%]
M5	~5	0	0	5.0	10.0	85.0
M5_L	~5	0	2.1	4.9	9.8	83.2
M5_L_RSV	~5	0.4	2.1	4.9	9.8	82.8
M8	~8	0	0	8.0	10.0	82.0
M8_L	~8	0	2.1	7.8	9.8	80.3
M8_L_RSV	~8	0.4	2.1	7.8	9.8	79.9
M10	~10	0	0	10.0	10.0	80.0
M10_L	~10	0	2.1	9.8	9.8	78.3
M10_L_RSV	~10	0.4	2.1	9.8	9.8	77.9

*PVA*, poly(vinyl alcohol); *RSV*, *trans*-resveratrol; *L*, Labrasol; *PG*, propylene glycol; *W* distilled water

First, PVA was dissolved in distilled water at 90°C under slow stirring. This solution was cooled down, and the amount of evaporated water together with 10% (*w/w*) of propylene glycol (PG) was added. The mixture was kept at room temperature (RT) overnight to remove air bubbles. Second, RSV was dissolved in Labrasol to obtain a stock solution of 15% (*w/w*). This stock solution was mixed with that of PVA, and the final concentration of RSV in the hydrogel was 0.4%. Third, the samples of 2.6 g were poured into a Petri dish of 28.5 mm in diameter, frozen at −80°C (Nuaire NU-9483E, Biogenet, Poland) for 24 h, and thawed at RT for 1 h. Then, they were subjected to six consecutive freeze-thaw cycles (6 FT). During one FT cycle, the samples were frozen at −80°C for 1 h, and thawed at RT for 1 h.

For reasons of comparison, six PVA cryogels without RSV were also fabricated (Table I), according to the procedure described above (the first step was followed by the third step).

### Morphology of Cryogel Membranes

#### Optical Microscopy

To study the morphology of the cryogel membranes, an optical microscope (Jenavert Carl Zeiss, Jena, Germany) equipped with a camera (Opta–Tech RT16 USB 3.0, Warsaw, Poland) was used. The samples were placed on a glass slide, and the images were recorded at the magnification of 100×.

#### Scanning Electron Microscopy

Prior to the analyses, the cryogel membranes were transformed into xerogels by freeze-drying (Alpha 2-4/LD, Martin Christ GmbH, Osterode am Harz, Germany). Then, the samples of xerogels were gently deposited on a conductive adhesive tape, previously glued to a specimen mount. A holder for nonconductive samples was used. The samples were analyzed using a Phenom Pro Desktop scanning electron microscope (SEM, PhenomWorld, Thermo Fisher Scientific, Waltham, MA, USA) equipped with a CeB_6_ electron source and a backscattered electron detector. The acceleration voltage was 10 kV, and the images were recorded at the magnification of 300×, 500×, 800×, and 1500×.

### Thermal Properties

DSC measurements were carried out using a DSC 821^e^ Mettler Toledo apparatus (Greifensee, Switzerland), equipped with an intracooler option (Haake, Karlsruhe, Germany). The samples, which weight was c.a. 3 mg, were placed in sealed aluminum pans. The lid of the pan was pierced to facilitate the evaporation of water and to prevent the crucible from breaking. An empty pan was used as a reference in all the measurements.

The following cooling–heating protocol was used in this study: (1) cooling ramp from 25 to −30°C; (2) isothermal at −30°C for 2 min; (3) heating ramp from −30 to 300°C. Either cooling or heating rate was 10°C min^−1^. The measurements were carried out in Ar atmosphere (60 mL min^−1^).

### Mechanical Properties of Cryogels

The mechanical properties of cryogel membranes were studied at room temperature (RT) using a tensile testing machine (Shimadzu EZ-SX, Kyoto, Japan) equipped with a 20-N load cell. The measurements were carried out according to the modified DIN EN ISO 527-2 standard as the size of the samples was reduced twice.

Samples in a specific dog bone shape (75 mm long, 10 mm wide at the ends and 5 mm wide in the middle) were prepared. Prior to the measurement, the thickness of each sample was examined at three different points using a digimatic micrometer (Mitutoyo 293-821, Kawasaki, Japan). Then, the samples were placed between two grips and the membrane was pulled by the top grip at the rate of 1000 mm min^−1^ up to the point at which the maximum force (F) was reached and the sample was ruptured.

Force, displacement, and time were measured directly. Stress (σ, Eq. 1), elongation (ε, Eq. 3), and Young modulus (E, Eq. 2) values were calculated, using a Trapezium X data processing software (Ver. 1.3.0 Shimadzu, Kyoto, Japan). Young modulus was calculated in the elastic behavior region while tensile strength (σF) corresponded to the maximum stress value obtained when the sample was ruptured (*n* = 6). All given values were referenced to the initial 55 mm gauge length of the specimens.

1 $$\upsigma =\mathrm{F}/\mathrm{A}$$

where, F was force [N], and A was the initial cross-section area of the specimen [mm^2^].

2 $$\mathrm{E}=\upsigma /\upvarepsilon$$

where ε was elongation calculated according to Eq. 3, ΔL was an increase in the specimen length [mm], and L_0_ was the initial length of the test specimen [mm].

3 $$\upvarepsilon =\Delta \mathrm{L}/{\mathrm{L}}_0\cdotp 100\%$$

### Assessment of pH

The value of pH of the cryogel membranes was measured using a pH meter (Mettler Toledo^TM^, S220, Greifensee, Switzerland). Prior to the measurements, the membranes of 5 g each were placed in bakers containing 50 g of distilled water (pH = 5.83 ± 0.12). They were incubated at 37°C for 24 h. To prevent the evaporation of water, Parafilm® M was used. The pH of PVA solutions before freeze-thawing cycling was also determined. Mean values (*n*=3) ± SD were calculated.

### Water Uptake Capacity

Gravimetric measurements were used to determine water uptake capacity (U_w_). The membranes were cut into 2 cm × 2 cm pieces, and the initial weight (W_d_) of the samples was recorded. Then, the samples were immersed in 0.9% NaCl solution and incubated at 37°C for 24 h. At scheduled time intervals, i.e., 10, 20, 30 min, and 1, 2, 3, 4, 5, 6, 7, 8, and 24 h; the samples were withdrawn, gently dried using a filter paper, and reweighted (W_s_). After weighing, the membranes were placed back in the solvent. Mean water uptake (*n*=6) in % ± SD was calculated according to Eq. 4 (*n*=6):

4 $${\mathrm{U}}_{\mathrm{w}}=\left({\mathrm{W}}_{\mathrm{s}}-{\mathrm{W}}_{\mathrm{d}}\right)/{\mathrm{W}}_{\mathrm{d}}\cdotp 100\%$$

where *W*_*s*_ was the weight of the swollen sample [g], and *W*_*d*_ was the initial weight of the sample [g].

### Magnetic Resonance Imaging

Multiecho pulse sequence (Multi Slice Multi Echo—MSME) was used to study the properties of cryogels. The samples were mounted in the dedicated non-magnetic holder which allowed nonrestricted swelling of the membrane. The holder was mounted in the cylindrical polycarbonate container filled with 24 mL of 0.9% NaCl and the container was placed in a 9.4-T Bruker Biospec MRI scanner (Bruker, Ettlingen, Germany). The studies were carried out at room temperature (~20°C). The following pulse sequence parameters were applied: field of view (FOV) = 30 × 30 mm^2^, slice thickness = 2 mm (axial cross section), number of slices = 5, slice separation = 3 mm, image matrix size = 128 × 128, echo time (TE) = 10.5 ms, number of echoes = 128, repetition time (TR) = 6.7 s, and number of signal averages (NA) = 1.

After immersion, the membrane swelling was assessed at 0.5, 24, and 48 h as an increase in the membrane cross-section area expressed in % of the membrane cross-section area measured before its immersion in water. The image segmentation was performed using a Trainable Weka Segmentation (TWS) plug-in module (http://fiji.sc/Trainable_Weka_Segmentation)-public domain software, Fiji distribution (http://pacific.mpi- cbg.de/wiki/index.php/Fiji). Two regions were assumed, i.e., the medium and the membrane. The swelling was calculated as an average of 5 slices ±SD (*n*=5) along the membrane.

For T_2_ relaxation time assessment, a small ROI was selected in the central slice. The image intensity was averaged over the selected ROI at every echo time. The resulting signal was fitted to the biexponential function using a MATLAB Curve Fitting Toolbox (MathWorks, Natick, MA, USA) by means of a Nonlinear Least Squares method, a Trust-Region algorithm.

### RSV Release from Cryogel Membranes

Franz diffusion cells were used to study the release of RSV from prepared cryogel membranes. They were cut into pieces of 2.5 cm × 2.5 cm to be mounted between a donor and a receptor compartment (*n*=3). The thickness of these membranes ranged from 3.25 to 3.42 mm. The diffusion area was 0.52 cm^2^. The donor compartment was covered with a Parafilm® M to minimize drying of the sample during the experiment. In order to ensure sink conditions, the receptor compartment was filled with 5 mL of PG. The study was carried out at 32°C. The solvent was stirred at 770 rpm with a magnetic bar. At scheduled time intervals, 0.5 mL of the sample was withdrawn from the receptor compartment, and the same volume of PG was added. The whole system was protected from the UV light exposure using a cover. The concentration of RSV in the samples was determined spectrophotometrically (Shimadzu UV-1800, Shimadzu USA Manufacturing Inc., Canby, OR, USA) at λ = 306 nm. The method was specific (Fig. S4). The calibration curve (Fig. S5) was linear from 4 to 40 μg/mL (r^2^ = 0.9999). The RSD (relative standard deviation) of intra- and interday precision, as well as that of accuracy at three concentrations (7.7 μg/mL; 16 μg/mL and 30 μg/mL), was lower than 5% (Tab. SI). Both model-dependent and model-independent kinetic parameters were calculated using *KinetDS v. 3.0*, an open source software (25).

## RESULTS AND DISCUSSION

Chemical properties, solubility, and crystallizability depend on the degree of hydrolysis, the molecular weight, and the content of acetate groups in PVA (10). The fully hydrolyzed grades of PVA are considered to be less soluble in water in comparison to those prepared of partially hydrolyzed grades. In this study, all tested cryogels were prepared using a high viscosity and the fully hydrolyzed PVA of a low content of residual acetyl. This grade dissolves in water forming clear and speck-free solutions at 90°C, is slightly soluble in ethanol (95%), but remains insoluble in organic solvents. Importantly, PVA is nonirritant to the skin at the concentrations up to 10% (26).

### Selection of Solvent for RSV

Due to the fact that RSV is poorly soluble in water (0.03 mg/mL at 37°C), the attempts were made to select a nonirritant solvent for this active ingredient that would be both compatible with the PVA hydrogel and suitable for a dermal application, especially in the wound bed area. The solubility of RSV was, therefore; analyzed in PG and Labrasol. The solubility of RSV in PG was 80.04 ± 1.14 mg/mL, but when PG was combined with water in 1:1 ratio, the solubility of RSV sharply decreased, and it was equal to only 5.34 ± 0.19 mg/mL. In contrast, the solubility of RSV in Labrasol was much higher than that recorded in PG alone, i.e., 140.80 ± 10.40 mg/mL. Since Labrasol (caprylocapryol polyoxylglycerides) is a solubilizing agent of self-emulsifying properties, it was assumed that its application could effectively prevent the precipitation of RSV while combining with an aqueous PVA solution. Labrasol was approved for the use in both cosmetic and pharmaceutical dermal formulations at high concentrations, namely, from 10 to 55% (26). Its suitability for the preparation of emulsions with either essential oils or antimicrobial agents for the therapy of wounds was also described (27). Apart from Labrasol, the review of the literature indicated that PEG 200, Cremophor RH 40, or Tween 80 could also be used as solvents for RSV (28–30). The solubility of RSV in these solvents was 30 mg/mL [RT], 150 mg/mL [37°C] or 80 mg/mL [37°C] respectively.

The images recorded immediately after mixing the stock solutions of RSV in PG, PEG 200, Labrasol, Cremophor R 40, or Tween 80, respectively, with the PVA sol are shown in Fig. S1. Regardless of the solvent, the combination of these two transparent or semitransparent solutions resulted in a significant increase in viscosity, and finally a semisolid, opaque, or even white system was formed. So high was the viscosity of the formulation prepared by the combination of PVA sol with RSV solution in PG or PEG 200 that it was impossible to pour it into a Petri dish (Fig. S1 a, b).

Better results were obtained when RSV was dissolved in Cremophor RH 40, Tween 80 or Labrasol as it was possible to pour the mixture into the glass molds. Yet, after freezing and thawing, the samples containing Cremophor RH 40 or Tween 80 became inhomogeneous (Fig. S1 c, d). Only when Labrasol was used as a solvent, the formulations remained uniform regardless of thermal processing (Fig. S1e). Therefore, RSV was dissolved in Labrasol to form the stock solution of 15% that was diluted with PVA sols, and then freeze-thawed.

### Morphology of Cryogel Membranes Loaded with RSV

The fabrication of hydrogel membranes that might serve as potential wound dressings should be optimized in such a way that will ensure high mechanical resistance together with a low risk of side effects related to the residues of chemical crosslinking agents (10). In this aspect, the application of repeated FT cycles to physically cross-link the polymer chains was an interesting alternative. However, the gel formation observed under repeated FT cycles was a complex phenomenon related to the crystallization of PVA and hydrogen-bond formation between the polymer chains, as well as liquid–liquid phase separation (31). All these phenomena determined the final microstructure of the cryogels (10, 13, 31, 32).

The impact of both concentrations of the matrix-forming polymer (PVA) and RSV loading on the appearance of cryogels prepared using FT is shown in Fig. S2. All *placebo* cryogels had smooth surface. They were colorless and their transparency depended on the concentration of PVA. The cloudiness, indicating the microphase separation related to the crystallization of PVA was observed while increasing the concentration of PVA from 5 wt.% to 8 or 10 wt.% (Fig. S2 a, c, e). By contrast to *placebo*, all cryogels loaded with RSV were milky-white and they had a smooth and a uniform surface (Fig. S2 b, d, f). Gupta *et al*. (33) who studied *placebo* PVA membranes, found a decrease in the transmittance of the samples with an increasing concentration of PVA. They also found positive linear correlations between the size of PVA crystallites and the concentration of PVA, as well as the number of FT cycles (33).

**Fig. 1:**
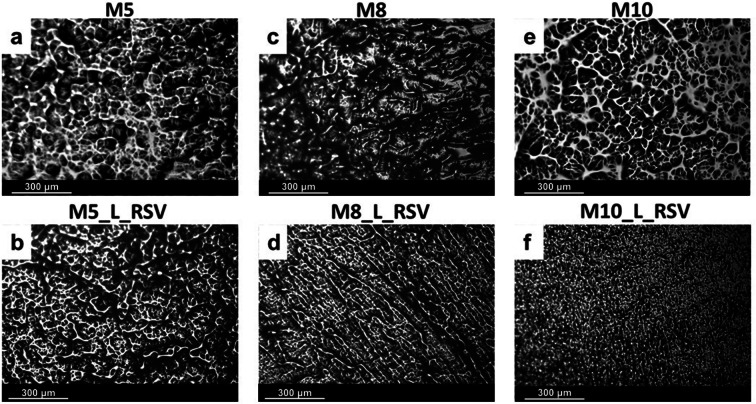
Images of cryogel membranes (M) captured using an optical microscope. First row shows *placebo* cryogels. Second row shows cryogels loaded with *trans*-resveratrol (RSV). Numbers correspond to percentage of PVA. L—Labrasol

**Fig. 2:**
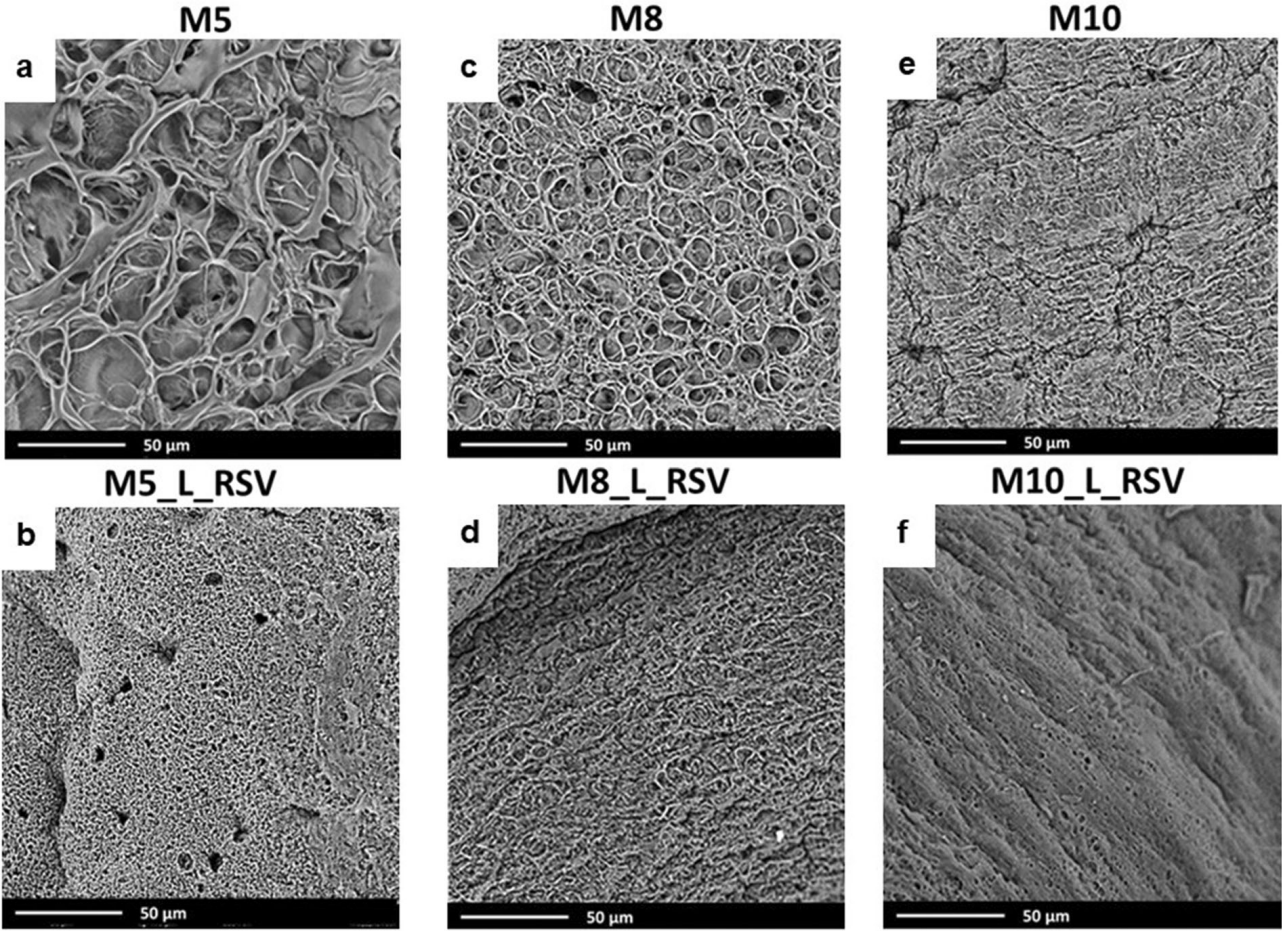
SEM pictures of freeze-dried cryogel membranes (M). First row shows *placebo* xerogels. Second row shows xerogels loaded with *trans*-resveratrol (RSV). Numbers correspond to percentage of PVA. L—Labrasol

The microstructure of cryogels was analyzed using both an optical microscopy and a scanning electron microscopy. The images are presented in Figs. 1 and 2, respectively. The images of cryogel membranes visualized using an optical microscope (Fig. 1) showed the segregation of a dense polymer skeleton from both water and solution phases typical of PVA hydrogels prepared by a repeated freezing and thawing procedure (13, 31). Despite the fact that three different concentrations of PVA were used to prepare *placebo* cryogels, the matrices looked similar (Fig. 1a, c, e). The loading of RSV into the matrix made the skeleton more dense and better organized, but the segregation was still visible (Fig. 1b, d, f). Interestingly, the concentration of PVA determined the shape of the solid network in cryogels loaded with RSV. The microstructure of M5_L_RSV (Fig. 1b) formed of 5% PVA was similar to that of *placebo* (Fig. 1a, c, e) . However, when the concentration of PVA rised from 5 to 8 wt.%, a dense solid fraction organized into a characteristic fibrillary pattern (Fig. 1d). A further increase in PVA loading up to 10 wt.% caused another reorganization of this solid network. As a result, a continuous skeleton made of a dense solid fraction was replaced by fine aggregates of a solid phase distributed homogeneously in the liquid phase of lower density (Fig. 1f).

The scanning electron micrographs of xerogels (freeze-dried cryogel membranes) showed that the composition of the matrix had an important impact on their internal structure (Fig. 2). The spaces left after the sublimation of frozen water upon freeze-drying reflected the size of ice crystals in the matrix. An increase in the concentration of the matrix-forming polymer (PVA) caused a gradual reduction in the pore size [M5 > M8 > M10] (Fig. 2). These observations were in line with the results of SEM studies described by Giusti *et al*. (34) who reported that the pore size decreased as the concentration and the number of FT cycles increased. In our studies, we found that the pore size in PVA xerogels decreased even more when RSV was loaded into the matrix, and finally, the microstructure of these xerogels was more compact than that of *placebo*.

### Thermal Properties of Cryogels

**Fig. 3:**
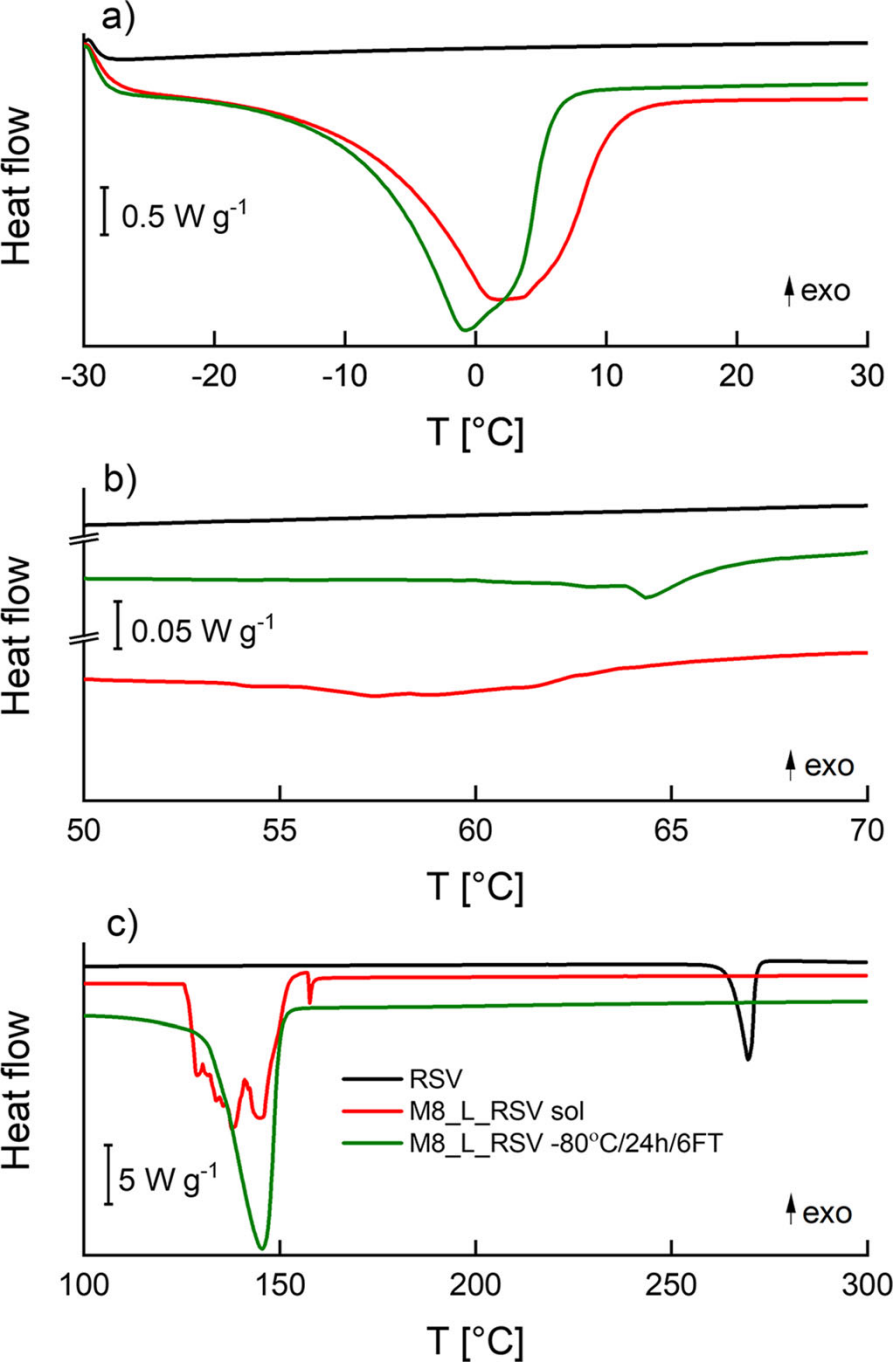
DSC curves recorded for RSV raw material (in black) and M8_L_RSV before freezing (in red), after freezing at −80°C for 24 h followed by 6 FT cycles (in green) in three temperature segments: **a** from −30 to 30°C, **b** from 50 to 70°C, and **c** from 100 to 300°C (10°C min^−1^)

The DSC curves recorded for RSV raw material (in black) and hydrogels composed of 8% of PVA loaded with RSV before (in red) and after a single freezing at −80°C/24 h followed by six consecutive FT (6FT) cycles (in green) are presented in Fig. 3. They correspond to the third part of the protocol used for DSC measurements (see MATERIALS AND METHODS section), in which the samples were heated from −30 to 300°C (10°C min^−1^). For better legibility, all heat flow curves were divided into three fragments, illustrating the main thermal phenomena typical of the tested cryogels, namely, (1) melting of solidified water (Fig. 3a), (2) melting of partially crystallized PVA (Fig. 3b), and (3) interaction of RSV with PVA (Fig. 3c).

The first of the aforementioned segments of DSC curves recorded from −30 to 30°C can be seen in Fig. 3a. In this range of temperatures, RSV exhibited no thermal events. As to either PVA sol or cryogels loaded with RSV, there was a wide asymmetric endotherm corresponding to the melting of solidified free water or bound water (35, 36). The onset of this endotherm slightly moved to lower temperatures after repeated FT cycles. Taking into account the enthalpy of melting, the amount of free water in hydrogels was estimated to be 53.8% and 50.2% for the hydrogel before freezing and the hydrogel freeze-thawed six times respectively.

The fragments of DSC curves recorded under heating of the samples from 50 to 70°C are shown in Fig. 3b. Similar to the heat flow curves presented in Fig 3a, RSV raw material did not show any thermal events, whereas in the DSC profiles of cryogels, small endothermic peaks typical of PVA could be seen (32). Before freezing, there was only an endothermic broadening in the DSC profile visible at the temperatures ranging from about 52 to 65°C. After six repeated cycles of FT, there was only one distinct endotherm with the onset at about 63°C and the minimum at about 64°C, providing the evidence that cooling of PVA hydrogel initiated the nucleation of PVA which could result in the growing strength of the final cryogel (13).

The melting of RSV could be seen as a single endotherm with the onset at 265°C in the first DSC curve from the top presented in Fig. 3c (in black). When RSV was combined with PVA hydrogel, a wide endotherm event at the range of temperatures from 125 to 155°C with multiple peaks followed by a small sharp endotherm with the onset at 157°C was visible (Fig. 3c in red). This wide endotherm may correspond to the melting of microcrystalline phases of PVA. After 6FT, there was only one wide endotherm with the onset at 130°C, indicating that after such a thermal treatment, the crystallites of PVA, and possibly also those of RSV were more homogeneous (Fig. 3c in green).

The full-range DSC profiles recorded for raw materials and all tested cryogels are presented in Fig. S3.

### Mechanical Properties

Wound dressings should have such a tensile strength that could withstand stresses exerted by the body. Additionally, they should be flexible enough to adapt to the wound bed topography and contour, preventing air- or fluid-filled pocket formation (31). On the other hand, they should leave no residue and show low adherence to the wound bed, reducing the risk of tissue damage when the dressing is removed.

**Fig. 4:**
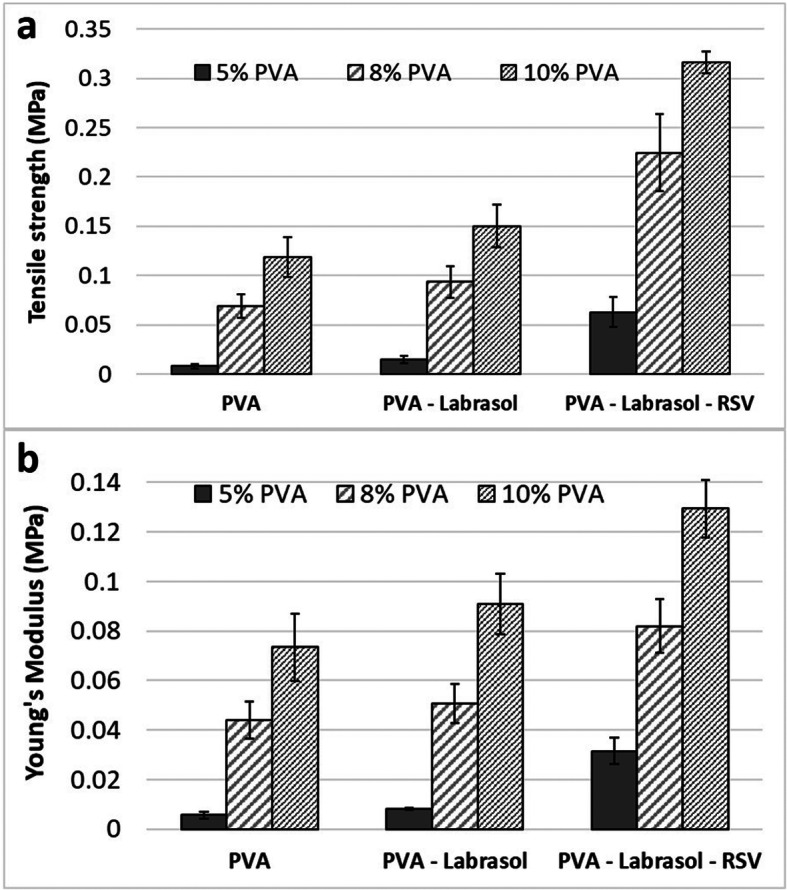
Impact of matrix composition on mechanical properties of cryogel membranes determined using a texture analyzer: **a** tensile strength; **b** Young modulus

In the present study, the impact of RSV on tensile strength and Young modulus (elastic modulus) of PVA cryogel membranes was studied (Fig. 4). According to our expectations, the values of these two parameters increased with the increasing concentration of the matrix-forming polymer—PVA (Fig. 4). The values of tensile strength were the lowest for cryogel membranes containing 5% of PVA. They were sticky, and they had a loose polymer network structure with the biggest spherical pores (Fig. 2). As a result, they could be damaged very easily. There was also a risk that they would adhere to the wound area, and in consequence, their removal may be painful. On the other hand, the cryogels which matrix was formed of 10% of PVA had the highest mechanical strength and they were the stiffest, which could make fixing them difficult. In contrast to the abovementioned formulations, it was found that the most favorable properties for the application in the wound bed had cryogels formed using 8% of PVA. These membranes were soft enough to adjust easily to the shape of the wound, but not sticky to strongly adhere. Thus, their mechanical resistance was the most suitable for comfortable and painless wound management.

After loading either Labrasol alone or RSV dissolved in Labrasol, an improvement in the stress resistance of all cryogles was stated (Fig. 4). The tensile strength of *placebo* membranes ranged between 0.02 ± 0.01 MPa for M5_L and 0.15 ± 0.02 MPa for M10_L, whereas after loading RSV, the tensile strength doubled or tripled, and it was equal to 0.06 ± 0.02 MPa for M5_L_RSV, 0.23 ± 0.04 MPa for M8_L_RSV, and 0.32 ± 0.01 MPa for M10_L_RSV.

The same was true when the flexibility of the membranes was evaluated using Young modulus. Adding RSV led to an increase in the values of this parameter in comparison to *placebo*. In the case of cryogels, containing 8% of PVA, the elastic modulus increased from 0.04 ± 0.01 MPa for M8, 0.05 ± 0.01 MPa for M8_L to 0.08 ± 0.01 MPa for M8_L_RSV, indicating high elasticity of cryogels loaded with RSV. They could be stretched up to 250% of their original length that was an important advantage, especially with regard to the treatment of burn wounds (37). These findings could be related to the less porous microstructure of the cryogels loaded with the solution of RSV in Labrasol as compared to those cryogels which did not contain RSV (Fig. 2). A fibrillary network typical of the cryogel M8_L_RSV (Fig. 1d) could be also responsible for a high flexibility of the membrane upon stretching. Similar relationship between the microstructure and the mechanical parameters were drawn by Zaman *et al*. (38) who studied gelatin/PEG membranes loaded with ciprofloxacin. In the presence of PEG, the flexibility of gelatin membranes improved as the system partially separated under processing and a loose network structure was created. The elongation at break for these membranes was higher than that of gelatin alone, but it was of only 40%. The impact of the microstructure on the mechanical properties of sodium alginate/PVA hydrogels matrices loaded with *Aloe vera* was studied by Bialik-Wąs *et al*. (39). The combination of sodium alginate with PVA improved the flexibility of brittle alginate dressings. The authors reported that microcrystals of PVA arranged into the fibrillary pattern, which enhanced the tensile strength and the flexibility of these membranes. Their elongation at break was of 70%, so the same as that of human skin (40), but still, it was much lower than that of M8_L_RSV cryogels (250%).

### Hydration-Related Phenomena

**Fig. 5:**
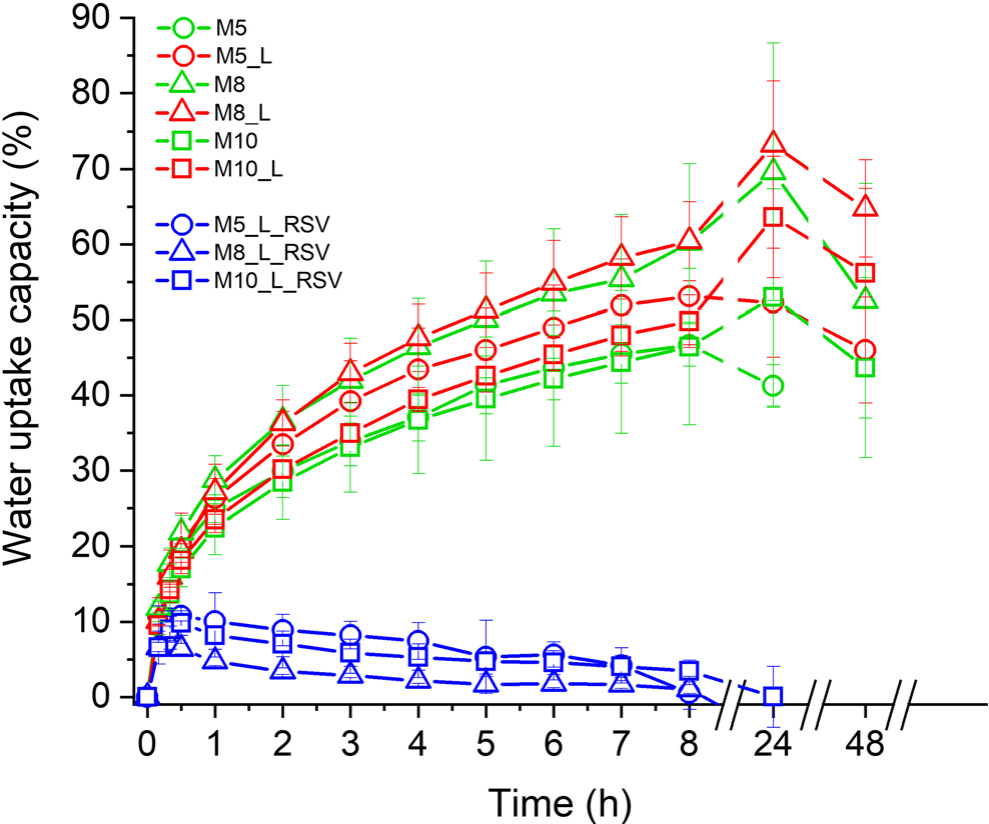
Impact of matrix composition on water uptake capacity (%) of cryogel membranes (*n* = 6, ± SD)

The ability to absorb water or exudate is an important feature of dressing materials as it can be helpful to clean the wound bed and to prevent recontamination. The hydrogel dressings display weak exudate absorptive capacity due to high water content (ca. 90 wt.%), but they can actively provide water to the wound bed. The rehydration of the wound enables the autolytic debridement of necrotic tissues, accelerating healing (5, 41). Therefore, the assessment of various phenomena related to the transport of water between the wound bed and the dressing material is recommended by compendia (37). Among them water uptake is widely used to evaluate absorbent properties of hydrogels. In this study, the impact of RSV on water uptake (U_w_) of PVA cryogels was studied, and the results of these gravimetric measurements in function of time are shown in Fig. 5.

In general, *placebo* membranes showed a gradual increase in water uptake for 24 h when the water uptake of 50% or 73% was recorded for the cryogels composed of 8 - 10% of PVA. Then, the reduction of this parameter to 40–65% was observed after 48 h. These findings could be attributed to the presence of soluble or mobile gel fraction that escaped from membranes (37).

Interestingly, the values of water uptake determined for cryogels containing Labrasol (in red) were slightly higher than those composed only of PVA and PG (in green). In turn, the loading of PVA hydrogels with the solution of hydrophobic RSV in Labrasol caused a significant decrease in water uptake (in blue) as compared to *placebo*. After 30 min, the cryogels loaded with RSV showed the maximal water uptake of only 10%. Then, a gradual decrease in the weight of cryogels was observed, and finally after 24 h, water uptake reached its initial values. Such a reduction in water uptake could be associated with a dense inner structure of cryogels loaded with RSV, visible in microscopic images (Figs. 1 and 2). The lack of space that could be filled with water and the presence of hydrophobic RSV in the cryogel matrix caused a significant reduction in water absorption. As it can be seen from Fig. 5, the concentration of the matrix-forming polymer—PVA—had lesser effect on water uptake than their loading with RSV.

**Fig. 6:**
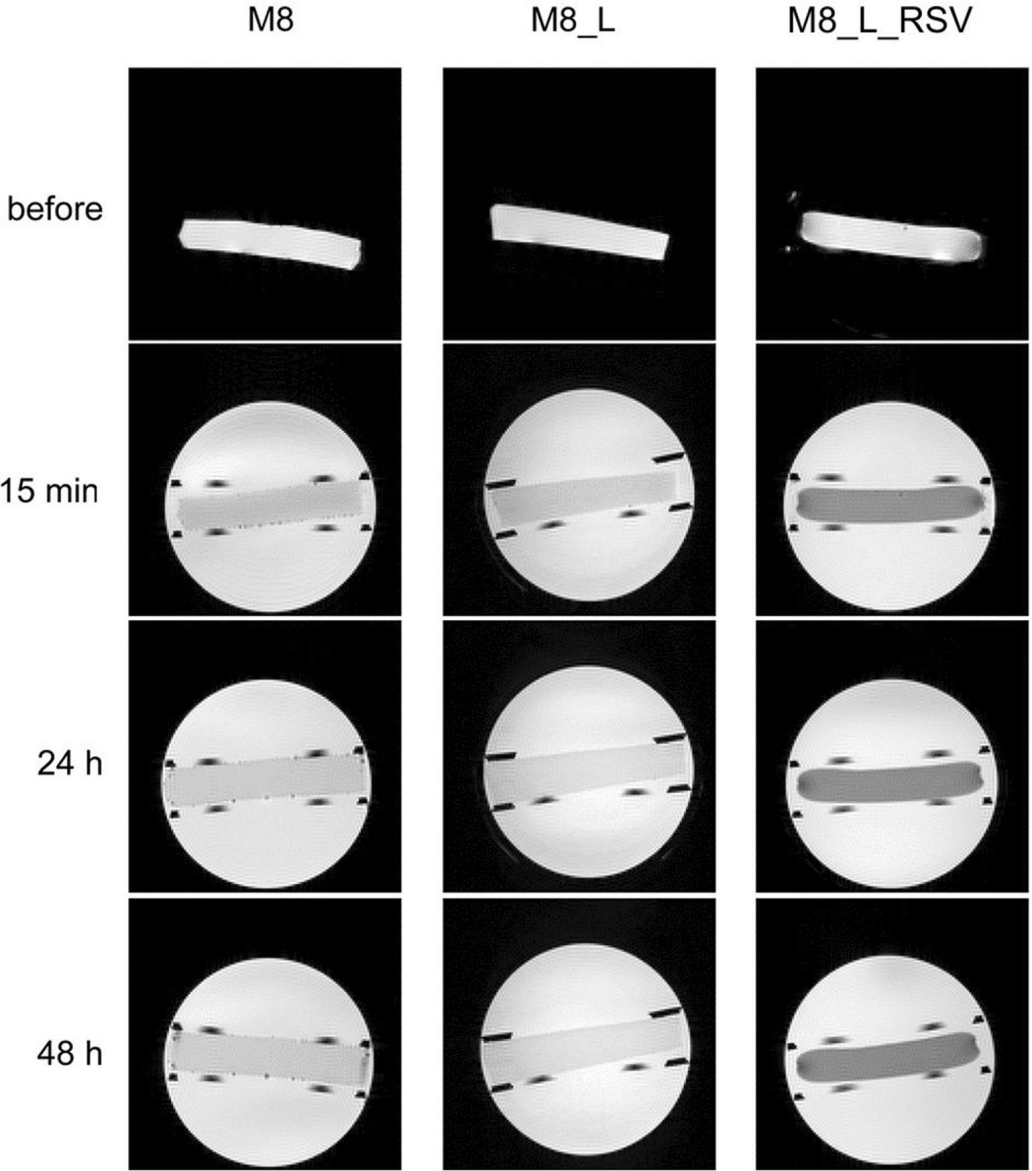
Selected MR images of cryogel membranes at various hydration times (TE = 10.5 ms)

**Fig. 7:**
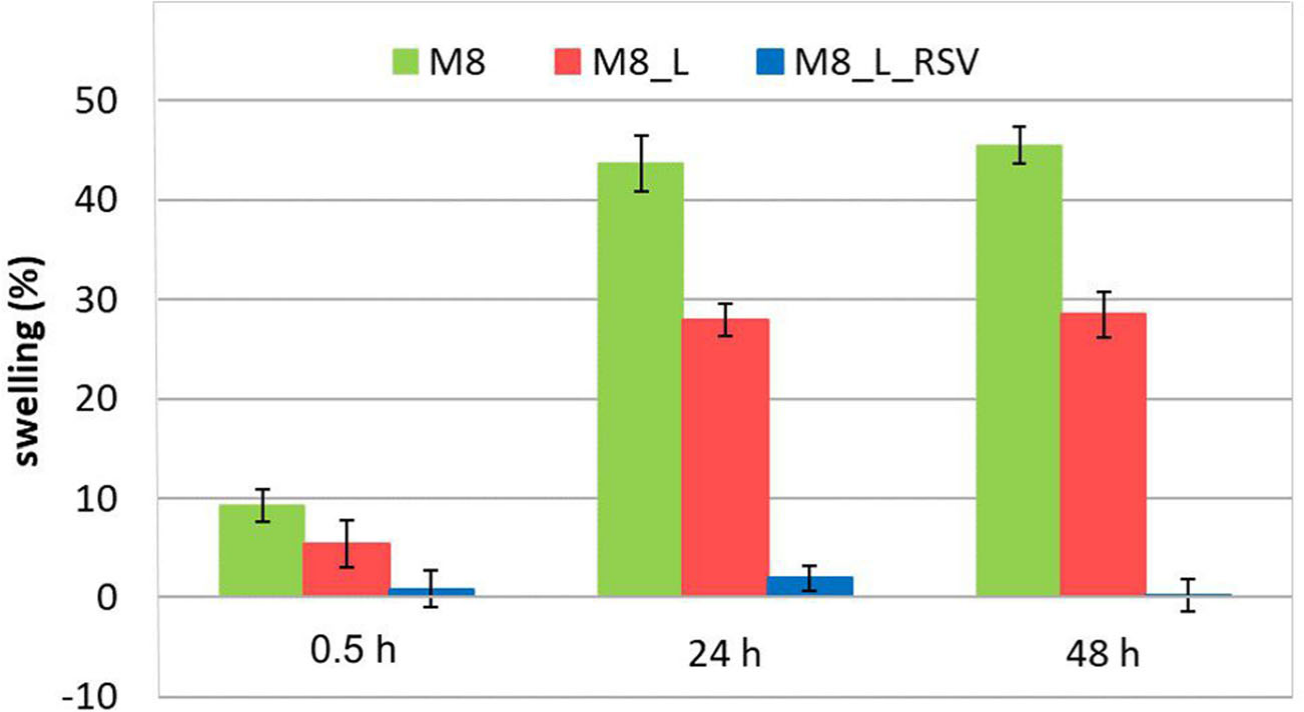
Swelling of cryogels: M8 (in green), M8_L (in red) and M8_L_RSV (in blue) on the basis of MRI data analysis

Taking into account the fact that the cryogels prepared using 8% of PVA had the most favorable mechanical properties for chronic wounds healing (i.e. high elasticity, low adherence), magnetic resonance imaging (MRI) was applied to study the swelling of these membranes. The results are presented in Figs. 6 and 7 and in Table II. The images presented in Fig. 6 show that cryogel membranes immersed in isotonic sodium chloride solution for 48 h preserved their shape and consistency—swollen membranes had sharp boundaries.

**Table II:** Results of swelling data analysis and multiecho data fitting for hydrogel membranes containing 8% PVA

Formulation	Hydration time (h)	Swelling (%)	SD	T_2s_ (ms)	95% confidence interval	T_2l_ (ms)	95% confidence interval	A_s_ (a.u.)	95% confidence interval	A_l_ (a.u.)	95% confidence interval
M8	0	0	-	41.84	41.56. 42.11	340.6	317.8. 363.4	1.050	1.047-1.053	0.071	0.069-0.074
	0.5	9.24	1.57	40.68	40.06. 41.3	216.1	194.3. 237.9	1.002	0.994-1.01	0.079	0.070-0.087
	24	43.66	2.78	86.62	86.27-86.97	-	-	0.986	0.983-0.989	-	-
	48	45.52	1.84	85.09	84.71-85.47	-	-	0.970	0.967-0.973	-	-
M8_L	0	0	-	28.37	27.93-28.8	236.6	223.2. 250	1.160	1.150-1.170	0.110	0.122-0.133
	0.5	5.38	2.37	38.22	37.16-39.27	152.5	134.9. 170.2	1.146	1.125-1.167	0.140	0.116-0.165
	24	27.97	1.61	67.04	66.42-67.65	-	-	1.130	1.122-1.138	-	-
	48	28.51	2.30	67.44	66.9-67.98	-	-	1.151	1.144-1.158	-	-
M8_L_RSV	0	0	-	22.38	21.15-23.6	121.3	108.2. 134.5	0.850	0.820-0.880	0.141	0.103-0.136
	0.5	0.84	1.89	30.00	26.6-33.4	108.70	72.78-144.6	0.943	0.886-0.999	0.132	0.0562-0.207
	24	1.90	1.29	40.07	39.08-41.06	-	-	0.823	0.807-0.838	-	-
	48	0.18	1.65	41.58	40.53-42.63	-	-	0.760	0.745-0.775	-	-

*SD*, standard deviation; *A*_*s*_, amplitude of shorter component; *T*_*2s*_, decay constant of shorter component; *A*_*I*_, amplitude of longer component; *T*_*2I*_*-*, decay constant of longer component

The spin-echo train envelope for membranes, before immersion in aqueous solution and immediately after immersion, had two distinct components, i.e. T_2s_ (shorter than 100 ms) and T_2l_ (longer than 100 ms). Taking into the account, the amplitude ratio, which should roughly reflect the concentration ratio (i.e., shorter to longer component amplitude ratio before immersion of A_s_/A_l_~0.1), it could be stated that the origin of a shorter component were water molecules and the origin of a longer component was the signal from propylene glycol. At 24 h and at 48 h of hydration, only one (shorter) component existed, indicating that propylene glycol leaked from the membrane (due to concentration gradient driven diffusion). The results of data analysis were specified in Table II.

According to the above mentioned, MRI relaxometry results could elucidate apparently anomalous (negative) water uptake in M8_L_RSV formulation (see Fig. 5). After the initial water uptake (~10% at 0.5 h of hydration) a gradual decrease in water uptake was observed during several hours down to ~0% at 24 h. Assuming that the long T_2_ component reflected the content of propylene glycol, the anomalous negative “water uptake” reflected the leakage of PG from the cryogel membrane (not a decrease in water content).

It was observed that the swelling degree changed with the membrane composition (Fig. 7). The cryogel membranes without RSV swelled extensively, starting from ~9% and ~5% at the first minutes after immersion up to ~43/45% and ~28% at 24/48 h of hydration for M8 (PVA), and M8_L (PVA-Labrasol) respectively. Together with this extensive swelling, T_2_ relaxation time also increased from 42 to 85 ms, and from 28 to 67 ms for M8 and M8_L, respectively.

In contrast, the cryogels containing RSV (M8_L_RSV) swelled only slightly, i.e., about 2% or less during 48 h of hydration, which corresponded to restricted water uptake (global hydration) as measured gravimetrically (Fig. 5). Despite the limited swelling of cryogel M8_L_RSV, T_2_ relaxation time increased from 22 to 42 ms after 48 h of hydration. It could suggest that there were several possible mechanisms of water T_2_ increase working together, e.g., an increase in the pore size during swelling and the lack of propylene glycol inside the membrane at 24/48 h. In the case of M8_L_RSV, the increase in T_2_ could be associated with propylene glycol removal only.

The MRI relaxometry results for membranes before immersion in 0.9% NaCl were related to the membrane morphology shown in SEM images of xerogels [lyophilized samples] (Fig. 2). M8 cryogel membrane after freeze-drying (xerogels) had ‘pores’ of roughly ~10 μm (see Fig. 2). The addition of Labrasol, as well as the addition of RSV dissolved in Labrasol, caused a reduction in the size of ‘pores’. Water T_2_ relaxation times (T_2s_) could reflect the confinement effect, i.e., the ‘pore’ surface relaxation. The values of T_2s_ were of 42 ms, 28 ms, 22 ms for M8, M8_L, and M8_L_RSV, respectively. It corresponded to the SEM microscopy where a decreasing “pore” size was observed in xerogels (from M8, M8_L to M8_L_RSV) and suggested the existence of pores in developed (nonfreeze dried) cryogels.

We consistently used the quantities introduced in our previous work (42), i.e., global hydration and swelling. The swelling term described changes in volume of the material during water uptake and the extent of swelling of a hydrogel was a balance between forces that constrained the network deformation and the osmosis that led to water absorption (43), while water uptake (we describe it also as global hydration) was a gravimetric parameter. Unfortunately, the terms such as swelling and water uptake are often used interchangeably, which can lead to serious misunderstandings.

The MRI studies supplied data complementary to water uptake results and SEM microscopy (Figs. 1 and 2). They allowed us to assess more precisely hydration related phenomena and provided some other hints, concerning the morphology of cryogel membranes.

### Impact of Formulation and Processing on pH of Cryogels

Human healthy skin has pH about 5.5, but the chronic wounds milieu is predominately alkaline, and it has tendency to shift toward acidic when the healing process begins. It was proven that alkaline environment could stimulate the growth of pathogens. Thus, the application of specialized wound dressings which would be able to modify the pH of the wound bed, may accelerate healing (44).

In this study, the pH of hydrogels was measured before and after freezing-thawing (Table III). The membranes *placebo* had the highest pH that ranged from 6.60 to 6.03. Interestingly, the pH of *placebo* PVA sols composed only of the matrix-forming polymer and PG was lower than that of FT cryogels, i.e., 5.41–5.62. Moreover, these values were similar to those recorded for *placebo* cryogels loaded with Labrasol or the stock solution of RSV in Labrasol. Importantly, the cryogels containing RSV had the lowest values of pH, i.e., below 5.27. This may be related to the fact that RSV is a polyphenol. Such a synergy between RSV and PVA in decreasing pH can be beneficial as it may give the opportunity to effectively control the pH of the wound bed.

**Table III:** Impact of formulation and processing on pH (*n* = 3, ± SD)

Formulation name	pH	
	PVA sols before FT	PVA cryogels after 6 FT
M5	5.62 ± 0.21	6.50 ±0.11
M5_L	-	5.27 ± 0.14
M5_L_RSV	-	5.26 ± 0.14
M8	5.47 ± 0.13	6.60 ± 0.17
M8_L	-	5.54 ± 0.02
M8_L_RSV	-	5.17 ± 0.02
M10	5.41 ± 0.17	6.03 ± 0.36
M10_L	-	5.24 ± 0.07
M10_L_RSV	-	5.27 ± 0.20

#### Release of RSV from Cryogel Membranes

Recently, there has been a growing interest in the design of PVA matrices that could not only effectively deliver antiinflammatory agents (45), antimicrobials (8, 46) or plant extracts (39) to the wound bed, but also control their release from the dressing material to this application site. Since the solubility of RSV in PG was 80 times higher than that determined in water or in buffers; PG was chosen to study its release from prepared cryogels. Such an approach ensured the maximal RSV concentration gradient between the tested membranes and the acceptor compartment (sink conditions).

**Fig. 8:**
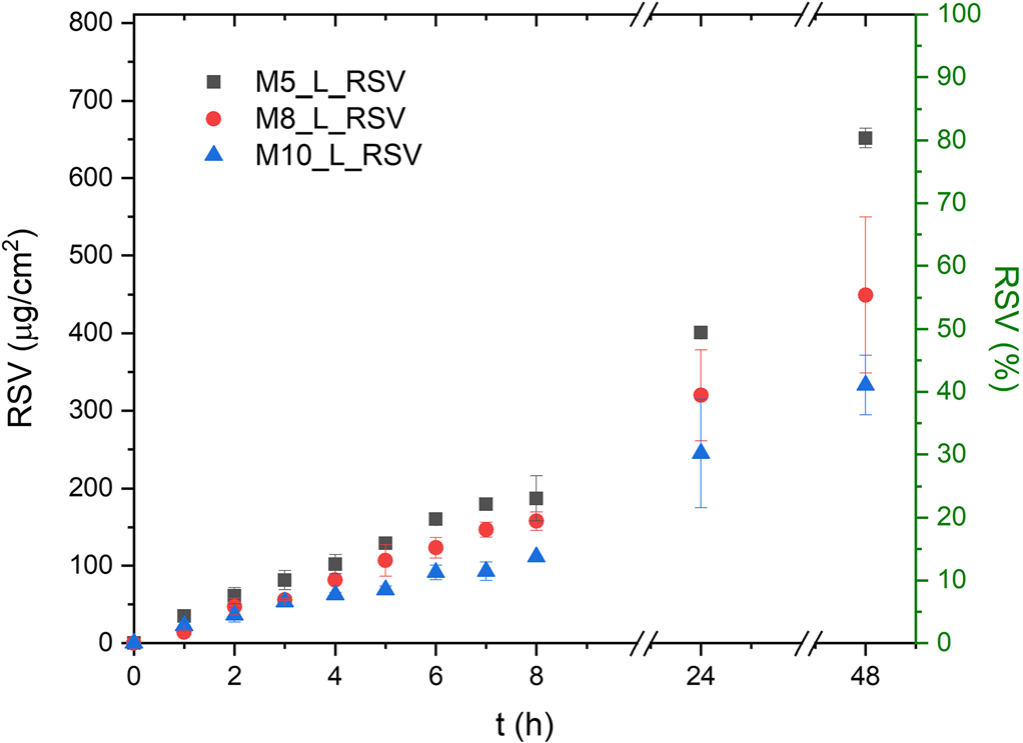
Impact of PVA content on release of resveratrol (RSV) from cryogel membranes (*n* = 3, ± SD)

As a rule of thumb, hydrogel dressings should be replaced every 1–3 days, depending on the hydration of the wound (41). Thus, in this study, the release of RSV was analyzed for 48 h. It was found that all tested cryogel membranes remained intact and there was no erosion visible even after 48 h of the test (see also the MR images presented in Fig. 6). However, the microscopic images of the cryogels (Figs. 1 and 2) showed that the higher the concentration of PVA was, the higher density of matrices was. In consequence, during the first 8 h of the study, only a small amount of RSV was released outside the matrices and the release rate was low (Fig. 8). The values of model-independent kinetic parameters, i.e., mean dissolution time (MDT) and dissolution efficiency (DE) shown in Table IV, increased from 17 to 20 h and from 27 to 46% with the concentration of PVA decreasing from 10 to 5%, respectively (Table IV).

**Table IV:** Model-dependent and model-independent parameters describing kinetics of RSV release from FT cryogels

Formulation	Model independent		Model dependent			
	MDT [h]	DE [%]	Korsmeyer–Peppas	Zero order	Higuchi	Hixson–Crowell
M5_L_RSV	20	47	K=10.30 *n*=0.31 r^2^=0.9581	K=1.62 r^2^=0.9741	K= 11.30 r^2^=0.8822	K=0.06 r^2^=0.6413
M8_L_RSV	18	35	K=7.66 *n*=0.30 r^2^=0.9262	K=1.14 r^2^=0.9339	K=8.45 r^2^=0.8912	K=0.05 r^2^=0.5959
M10_L_RSV	17	26	K=6.24 *n*=0.29 r^2^=0.9562	K=0.84 r^2^=0.9445	K=6.92 r^2^=0.7802	K=0.05 r^2^=0.6152

*MDT* mean dissolution time, *DE* dissolution efficiency, *K* dissolution rate constant, *n* Fickian release exponent, *r*^*2*^ empirical coefficient of determination

The values of model-dependent kinetic parameters gathered in Table IV showed a good fit to the semiempirical model of Korsmeyer–Peppas with r^2^ ranging from 0.9262 to 0.9581. The values of the Fickian exponent (n) were close to 0.3, indicating that the release of RSV from the tested cryogels relied mainly on diffusion. Similar values of r^2^ were found for the zero order model, providing the evidence that PVA matrix was able to effectively control the release rate of RSV. When Higuchi model was investigated, r^2^ were below 0.9. Finally, the lowest r^2^ were stated for Hixson-Crowell model, which also supported the conclusion that erosion was not involved in the release of RSV, which was in agreement with our previous observations using MR imaging.

The suitability of PVA hydrogels to control the release of drugs was well documented (47–49). The mechanism of drug release from FT cryogels might rely on the interactions between the solvent and the amorphous regions in the semicrystalline polymer network. In consequence, the size of the crystallites formed under repeated FT could determine the release rate (10). On the other hand, some studies showed that the microporous structure of the system in which supramolecular and semicrystalline regions coexisted could be the main determinant responsible for controlled release (49).

In this study, all cryogels were prepared using the same processing (6FT). The most important structural rearrangements were observed while loading RSV into the PVA sol (Fig. S1) and after early freezing-thawing. They might be related to the hydrogen bonding between phenolic groups of RSV and hydroxyl groups of PVA (50). This chemical interaction together with the physical cross-linking between PVA crystallites may cause a significant densification of matrices visible in Fig. 2. When the concentration of the matrix-forming polymer increased, the number of interactions on the PVA-RSV interface also increased, which was finally observed as a sustained RSV release. As expected, this effect was the most pronounced for cryogels of the highest content of PVA, i.e., 10% (Fig. 8).

### Summary of Advantages and Drawbacks of Developed Cryogels

Despite the fact that there is a great variety of wound dressings available on the market, the wound management is still challenging due to the dynamics of the healing process. For this reason, there is no perfect dressing suitable for every wound as different materials have to be used when healing progresses. The major advantage of modern hydrogel dressings is the ability to maintain the appropriate level of moisture in the wound bed and to protect it from further injuries. Currently, the development of active hydrogel dressings, loaded with antibacterial or anti-inflammatory compounds that could accelerate healing is of particular interest (51).

Taking into account self-healing, as well as biomimetic properties of PVA (18), the opportunity to form hydrogels under physical cross-linking, the ability of such hydrogels to control the release of drugs (49), this polymer was chosen to form cryogel membranes from which RSV could be released for several hours. Since RSV is poorly soluble in water, it was necessary to dissolve it in Labrasol, and in this form combine it with PVA sols. Finally, cryogel membranes were successfully prepared by freezing and thawing. However, their microstructure was very dense. The fibrillary arrangement of the polymer network resulted in high tensile strength and flexibility much higher than that of the human skin. These features were beneficial as low tensile strength is one of the most important drawbacks of hydrogel dressings (51). Moreover, the report of Comotto *et al*. (52) showed that when RSV was loaded in the matrices formed of alginates, the increase in tensile strength was insignificant (52). Thus, the combination of RSV with PVA in the freeze-thawing process enabled to overcome this disadvantage.

On the other hand, our research also showed that such a dense PVA matrix loaded with RSV had a negative impact on water uptake, estimated using the gravimetric method. These findings were also confirmed by reduced values of T2 found in MRI relaxometry. Yet, taking into account that hydrogel dressings are generally recommended for the treatment wounds with low or moderate exudate, these results could be acceptable (37). Importantly, the release studies confirmed that RSV diffused from prepared matrices for 2 days.

All in all, the manufacturing of active hydrogel dressings loaded with poorly soluble active ingredients could be challenging, especially if the membranes of high mechanical resistance, high water uptake and controlled release properties are needed.

## CONCLUSIONS

In this study, we successfully developed and thoroughly characterized hydrophilic PVA cryogel membranes loaded with a poorly soluble active ingredient of antiinflammatory properties—RSV. Bearing in mind that these hydrogel membranes would be applied in the wound bed, a freeze-thawing technique was used to transform PVA sol into gel. In such a way, there was no need to use any chemical cross-linking agents, which residues could cause irritation. Among three tested concentrations of the matrix-forming polymer, the best mechanical properties and elasticity showed cryogels prepared using 8% of PVA. In comparison to *placebo*, the cryogels loaded with RSV were less porous and showed higher mechanical strength. The lack of big pores and a dense cryogel network formed in the presence of RSV resulted in low water uptake capacity of these membranes. Interestingly, RSV incorporated into PVA cryogels showed a synergy in lowering the pH of FT membranes, which could accelerate the wound healing process. Furthermore, a nonerodible PVA matrix formed under repeated FT cycles was able to control the diffusion of RSV for 48 h.

## Supplementary Information

ESM 1 (DOCX 1562 kb)
