# Supplementary material for: Poly(Vinyl Alcohol) Cryogel Membranes Loaded with Resveratrol as Potential Active Wound Dressings
Source: AAPS PharmSciTech. 2021 Mar 14;22(3):109. doi: 10.1208/s12249-021-01976-1 (PMC7956935; doi:10.1208/s12249-021-01976-1)
Supplement: Supplementary file 1 — (DOCX 1562 kb) [file 12249_2021_1976_MOESM1_ESM.docx]

**Supplemental**

Poly(vinyl alcohol) cryogel membranes loaded with resveratrol as potential active wound dressings

Anna Górska^1^, Anna Krupa^1^*, Dorota Majda^2^, Piotr Kulinowski^3^, Mateusz Kurek^1^, Władysław P. Węglarz^4^, Renata Jachowicz^1^

**
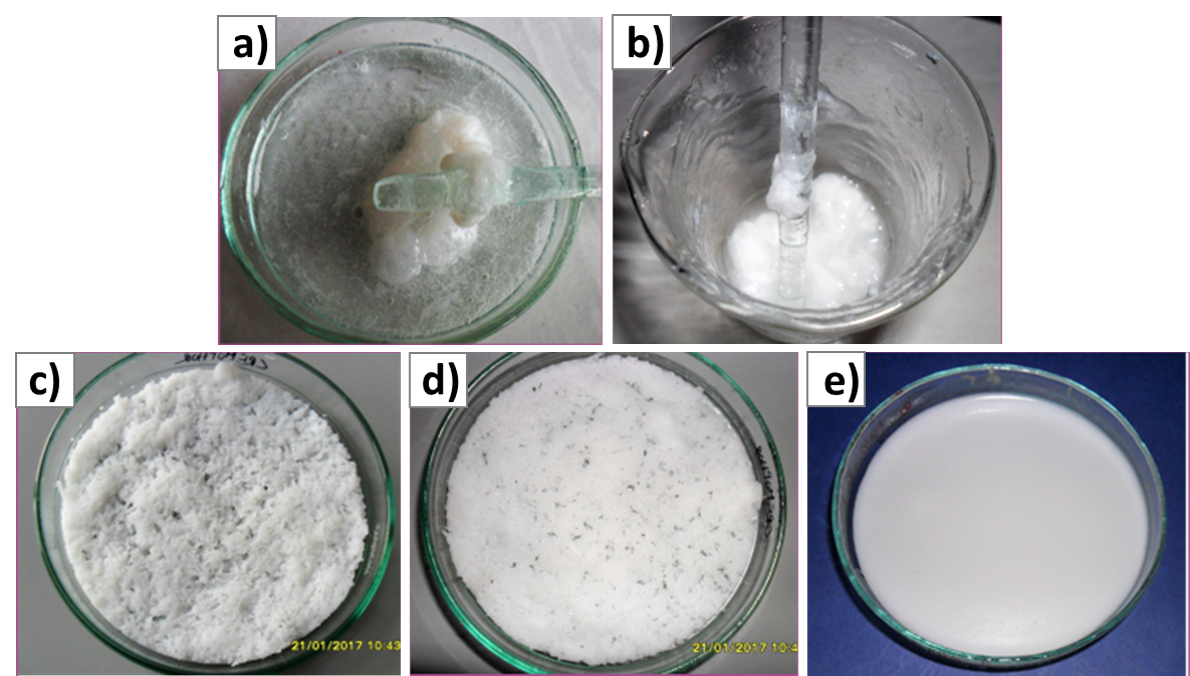
**

**Fig. S1.** Images taken immediately after combining PVA sols of 8 % with 15 % solution of RSV in: a) PG, b) PEG 200 or after freezing and thawing c) Cremophor RH 40, d) Tween 80, e) Labrasol.


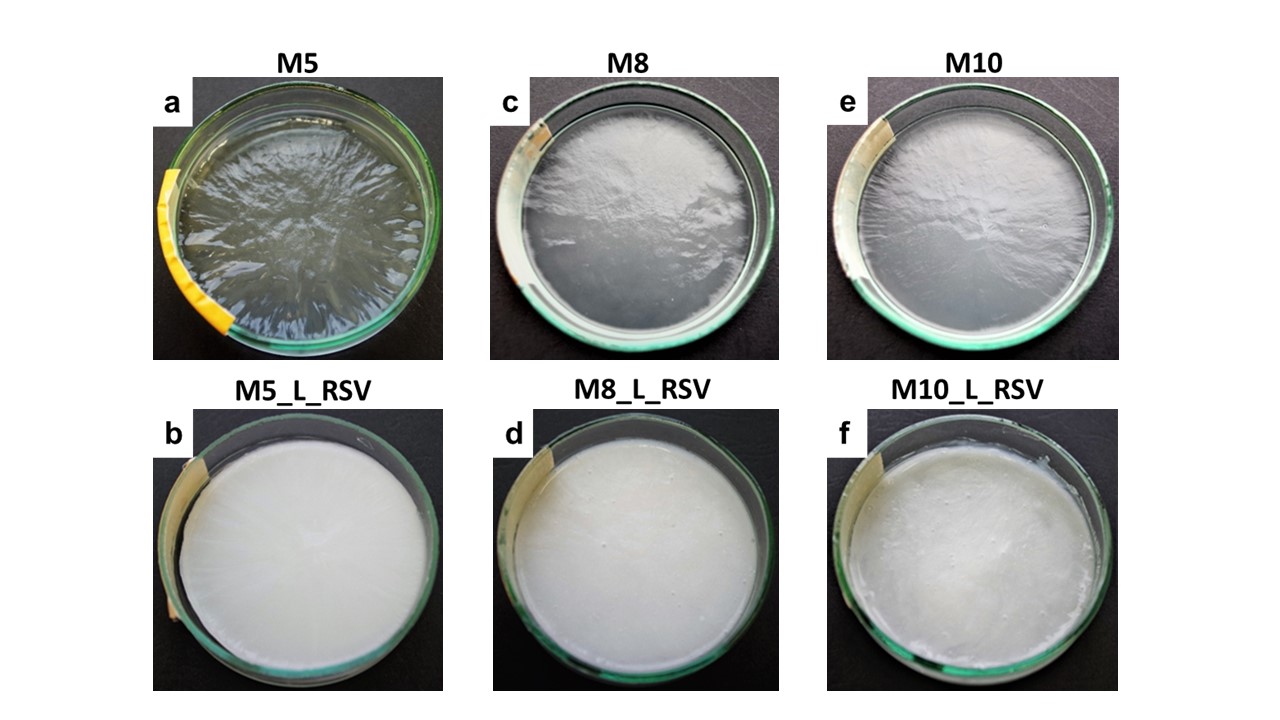


**Fig. S2.** Images of cryogel membranes (M) recorded after 6FT cycles using digital camera. First row shows *placebo* cryogels. Second row shows cryogels loaded with *trans*-resveratrol (RSV). Numbers correspond to percentage of PVA. L-Labrasol.

**

**

**Fig. S3.** Heat flow curves of: (a) raw materials: *trans*-resveratrol (RSV), polyvinyl alcohol (PVA), propylene glycol (PG), Labrasol (L), stock solution of 15 % RSV in L, sol M8 (PVA & PG), sol M8_L (PVA, PG & L), sol M8_L_RSV (PVA, PG, L & RSV); (b-d) M8, M8_L and M8_L_RSV before and after 6FT cycles.

**Fig. S4**. Specificity of RSV quantification in cryogel membranes using UV spectroscopy.

**Fig. S5.** Calibration curve of RSV in propylene glycol determined at λ = 306 nm (r^2^ = 0.9999).

**Tab. SI.** Intra- and interday precision of RSV measurements (amber glass).

| Samples | | RSV [μg/mL] | | | | |
| --- | --- | --- | --- | --- | --- | --- |
|  |  | 0 min | 24 h | 48 h | 72 h | 96 h |
| A  $\bar{x}$ [μg/mL] = 7.74  SD [μg/mL] = 0.05  RSD [%] = 0.65 | 1 | 7.72 | 7.77 | 7.78 | 7.74 | 7.65 |
|  | 2 | 7.74 | 7.74 | 7.72 | 7.74 | 7.67 |
|  | 3 | 7.74 | 7.87 | 7.73 | 7.75 | 7.68 |
| B  $\bar{x}$ [μg/mL] = 15.98  SD [μg/mL] = 0.04  RSD [%] = 0.25 | 1 | 15.97 | 16.03 | 15.98 | 16.05 | 15.92 |
|  | 2 | 15.95 | 16.03 | 15.98 | 15.99 | 15.95 |
|  | 3 | 15.96 | 15.98 | 15.92 | 16.00 | 16.04 |
| C  $\bar{x}$ [μg/mL] = 30.40  SD [μg/mL] = 0.13  RSD [%] = 0.43 | 1 | 30.22 | 30.23 | 30.39 | 30.65 | 30.54 |
|  | 2 | 30.22 | 30.34 | 30.39 | 30.49 | 30.44 |
|  | 3 | 30.26 | 30.42 | 30.42 | 30.47 | 30.51 |
